# Impact of food insecurity and food environment on the diet quality of older African Americans during the COVID-19 pandemic

**DOI:** 10.3389/fpubh.2023.1268961

**Published:** 2023-11-14

**Authors:** Lucy W. Kibe, Katrina Schrode, Mohsen Bazargan, Magda Shaheen

**Affiliations:** ^1^Physician Assistant Program, Charles R. Drew University of Medicine and Science (CDU), Los Angeles, CA, United States; ^2^Department of Psychiatry, Charles R. Drew University of Medicine and Science (CDU), Los Angeles, CA, United States; ^3^Department of Family Medicine, Charles R. Drew University of Medicine and Science (CDU), Los Angeles, CA, United States; ^4^Department of Family Medicine, University of California, Los Angeles (UCLA), Los Angeles, CA, United States; ^5^Department of Internal Medicine, Charles R. Drew University of Medicine and Science (CDU), Los Angeles, CA, United States

**Keywords:** COVID-19, diet quality, food insecurity, food environment, African American, older adults, healthy eating index

## Abstract

**Introduction:**

A high quality diet is vital in promoting wellbeing and ensuring good health, particularly for those living with chronic conditions. Older African Americans, already burdened with a higher prevalence of chronic conditions, also face a higher risk for suboptimal diets. The COVID-19 pandemic had lasting effects on access to healthy food for all Americans, but some demographic groups were disproportionately affected. Older African Americans, who already experienced reduced access to healthy food pre-pandemic, were particularly afflicted, but the full extent of the pandemic's impact on their food insecurity and food environment remains unclear.

**Methods:**

To address this gap, we conducted a study among 102 older African Americans in South Los Angeles between October 2021 and July 2022 during the COVID-19 pandemic. Participants completed surveys on dietary intake, food insecurity, and neighborhood food environment. We measured dietary quality using the healthy eating index (HEI)-2015. The analysis included descriptive, bivariate chi-square, *t*-tests, analysis of variance, and multiple linear and logistic regression.

**Results:**

While overall dietary quality was suboptimal, most participants met the guidelines for fruit and vegetable consumption. Food insecurity was associated with lower overall diet quality and lower total fruit and whole fruit intake. However, there was no association between food environment and diet quality.

**Discussion:**

In light of our findings, further intervention is critical to improving diet quality, especially among older African Americans living with chronic conditions in the post-pandemic era.

## 1. Introduction

Chronic conditions such as heart disease, diabetes, and hypertension continue to rank as leading causes of morbidity and mortality among older Americans (1). There is well-established evidence that a high-quality diet plays an important role in preventing or slowing these diseases and promoting overall wellbeing (2–5). This is particularly important among older African Americans, who are disproportionately burdened with chronic conditions (1) and have historically reported suboptimal diets based on recommended guidelines (6–10). Prior studies suggest that access to healthy food significantly influences in diet quality (11–15). However, it remains unclear how changes in the experiences of food insecurity and the food environment impacted the diet quality of older African Americans during the COVID-19 pandemic. This is of considerable importance because chronic diseases may be exacerbated by diet changes experienced during this pandemic.

Food insecurity is a condition in which an individual lacks consistent access to enough nutritious food to live an active and healthy life (16). It is often caused by a lack of financial resources or inadequate access to healthy and affordable food options. Food insecurity is associated with poor diet quality, prevalence of chronic disease, and higher mortality rates (17–21). On the other hand, the food environment refers to the physical environment that influences access to healthy food options in a particular area or community (15). It includes factors such as the availability and affordability of high quality food, as well as the types of food outlets (such as grocery stores, convenience stores, fast food restaurants, and farmers' markets) present in the area. Particularly for low-income and minority communities, limited access to affordable and nutritious food options may significantly impact dietary habits and health outcomes (22–24).

A longitudinal study by Leung et al., (19) found a positive relationship between food insecurity and poor diet quality among adults over the age of 60 years. Furthermore, a systematic review and meta-analysis of existing literature by Gundersen and Ziliak (20) demonstrated that food insecurity was consistently associated with worse physical and mental health outcomes, including chronic diseases. The link between food insecurity and chronic diseases such as heart disease, diabetes, and hypertension was also observed among low-income participants in the National Health and Nutrition Examination Survey (NHANES) (21). Recognizing the negative impact of food insecurity on disease outcomes is critical now more than ever, considering the escalation of food insecurity during and post the COVID-19 pandemic (25, 26).

The COVID-19 pandemic exerted a disproportionate impact on food environments and food insecurity, particularly among vulnerable populations such as older adults, low-income individuals, and minorities (17, 25, 27–32). Notably, Dubowitz et al. (28) reported that among African American residents in a low-income food desert neighborhood, food insecurity increased from 21% in 2018 to 36% in 2020 as a result of the pandemic. Additionally, several studies quantified the number of food outlet closures in locations around the US (33–35). In Flint, MI, 173 food venues closed during the pandemic, with only 17 new venues taking their place (33). Locations with predominantly African American populations had three times the rate of emergency food outlet closures as other locations (33). Similarly San Diego county lost a net of 8% of its stores that accept electronic benefits transfer (EBT) payments, including one full-service supermarket (35). These closures, coupled with supply-chain shortages and business restrictions, significantly impacted access to healthy food options (31, 32, 36). The repercussions of these changes in the food landscape pose substantial challenges to vulnerable communities already facing food insecurity.

Recognizing the potential compromise to diet quality during the pandemic and the subsequent potential impact on the health status of older African Americans, our study aimed to examine the effects of food insecurity and food environment on their diet quality. Our hypothesis posited that (1) a lower food insecurity would be associated with higher diet quality, and (2) a favorable food environment would be associated with higher diet quality.

## 2. Materials and methods

### 2.1. Participants

The COVID-19, Food insecurity, Exercise, and Dietary history (C-FED) study was a cross-sectional sample of a larger intervention study focused on health behaviors in older African Americans in South LA during the COVID-19 pandemic (37). To be eligible for the parent study, participants had to identify as Black or African American and be at least 65 or 55 years old with a chronic condition. All participants enrolled in the parent study were eligible to take part in the C-FED study. A total of 118 participants opted to join the C-FED study and complete the study-related surveys between October 2021 and July 2022. Consistent with previous studies, we excluded individuals whose responses to the diet history questionnaire indicated improbable caloric intake (<500 kcal/day or >3,500 kcal/day for women; <800 kcal/day or >4,000 kcal/day for men) (38–40), resulting in an analytical dataset of 102 participants. Informed consent was obtained prior to their participation. The C-FED study was approved by the Charles R. Drew University of Medicine and Science IRB.

### 2.2. Surveys

Details on the methodology and design of the C-FED study are presented in a previous manuscript (37). Participants completed surveys on their own, through an online link, over the phone with a trained research assistant, or in person with a trained research assistant. The C-FED surveys consisted of the Diet History Questionnaire (DHQ) III, the National Center for Health Statistics six-item short form food security survey, and three questions about food environment. Socio-demographic and health information were obtained from the parent study questionnaires. Socio-demographic information included age, gender, education level, income, and living arrangement. Education level was categorized into three groups: high school degree or less, associate degree or some college, and college or post-graduate degree. Annual income was dichotomized using a cutoff of $50,000. For living arrangement, participants were asked if they lived alone (yes/no). Health information consisted of self-reported health, BMI, and chronic conditions. Health rating options included poor, fair, good, very good, and excellent. We combined groups with small numbers, resulting in the categories poor-fair, good, and very good-excellent. BMI was categorized according to clinical guidelines into healthy (18.5–24.9), overweight (25.0–29.9), and obese (≥30). We constructed the variable number of chronic health conditions by counting the number of health conditions that participants listed. This variable was categorized into 0, 1, or 2, and 3+ health conditions.

### 2.3. Food insecurity

The food insecurity questionnaire reflects household level food insecurity. This measure has been previously validated as correctly classifying 97.7% of households in the U.S. (41, 42). It consists of six questions that ask about running out of food and skipping or cutting the size of meals. The total food insecurity score is calculated as the total number of items endorsed by the participant, such that the total possible score ranges from 0 to 6. A score of 0 indicated full food security, while scores ranging from 1 to 6 indicate increasing levels of food insecurity. We used a cutoff of 1 to dichotomize this variable into those with food insecurity (code = 1) and without food insecurity (code = 0).

### 2.4. Food environment

We asked three questions related to the food available in the neighborhood taken from the PhenX Toolkit (https://www.phenxtoolkit.org/protocols/view/210701). An assessment of these questions reported an internal reliability of 0.78 and test-retest reliability of 0.69 (43). Participants were instructed to consider their neighborhood as within 1 mile of their home. Participants were asked to rate their level of agreement with (1) The fresh fruits and vegetables in my neighborhood are of high quality, (2) A large selection of fresh fruits and vegetables is available in my neighborhood, and (3) A large selection of low-fat products is available in my neighborhood. Responses were on a 5-point Likert scale ranging from strongly disagree to strongly agree. Following instrument guidelines, we created an overall food environment score by taking the average of the scores of the three questions and rounding to the nearest integer. All food environment variables were categorized into three groups: disagree (scores <3), neutral (score = 3), and agree (scores > 3).

### 2.5. Outcome variables: diet quality

The DHQ is a comprehensive diet inventory. Participants were asked about the frequency and quantity of all types of foods consumed over the past 12 months. Diet quality was measured using the Healthy Eating Index-2015 (HEI), which was calculated based on responses on the DHQ. The HEI is a validated construct for assessing dietary intake (44). It consists of the sum of 10 sub scores, each of which reflect consumption of a particular food group or food category. A key feature of HEI is that scores are based on density, so it reflects the balance of food consumed across food groups. Thus, the total score reflects diet quality, as opposed to quantity.

The total score ranges from 0 to 100, with 100 reflecting the ideal diet according to the dietary recommendations of the USDA. The USDA suggests the following grading for total HEI scores: 90–100 = A; 80–89 = B; 70–79 = C; 60–69 = D; <60 = F (45). In addition, the score for overall HEI was normally distributed using the Shapiro-Wilk test and used in the analysis as continuous variable.

We focused on the diet quality indices that correspond to the neighborhood food environment questions: HEI sub scores for total fruit, whole fruit, vegetable, percent of calories from total fat, and percent of calories from saturated fat. Total fruit score includes 100% juices, while the whole fruit score includes all forms of fruit except juice. All the included HEI sub scores range from 0 to 5, with 5 indicating highest quality. Because the distributions of the HEI sub scores were highly skewed, we categorized these variables into those who achieved the maximum possible score (5) and those who did not (<5). The percentage of calories consumed from fat and from saturated fats were normally distributed per Shapiro-Wilk Tests and treated as continuous variables.

### 2.6. Statistical analysis

Descriptive statistics were used to depict the population characteristics. Categorical data were presented as number and percent. Continuous data were presented as mean and standard deviation for normally distributed variables and median and inter quartile range for skewed variables. For bivariate analyses, we used chi-squared tests to test for statistical significance between categorical variables, and *t*-test or analysis of variance (ANOVA) for associations between categorical and continuous normally distributed variables. We used multiple regression to test for statistical associations of food insecurity and food environment with diet quality. We used the overall food environment score in multiple regressions and controlled for variables that could be associated with diet quality: age, gender, education, living arrangement, physical health, BMI, and number of chronic conditions. Age was included in the model as a continuous variable; the other variables were included as categorical variables. To avoid multicollinearity, we did not include income in the models, due to its strong correlation with food insecurity. Our small sample size did not provide us with the power to investigate interactions.

The diet quality outcomes included total HEI, HEI sub scores of fruit, whole fruit, and vegetables, and percent of calories from fats and from saturated fats. Linear regression was used where the dependent outcome was a continuous normally distributed variable (total HEI, percent calories from saturated fat, and percent calories from fat), and logistic regression was used for categorical binary outcomes (HEI sub scores of fruit, whole fruit, and vegetables). For linear regressions, we present adjusted B coefficient and standard error (SE); for logistic regressions we report adjusted odds ratio and 95% confidence interval. The percent missing for any given variable was <5%, except for education level which was missing for 8%. We used listwise deletion for cases with missing data in multivariable regressions. Analyses were done using SAS 9.4, and a *p* < 0.05 was considered statistically significant.

## 3. Results

### 3.1. Participant characteristics

Participant ages were nearly evenly distributed (Table 1). Most participants were female (71%), did not live alone (70%), and had relatively low incomes with 75% reporting an annual income <$50,000. Thirty-five percent had an education level of high school degree or less, while 23% had a college or post-graduate degree. While 65% rated their health as very good, good, or better excellent, 76% were overweight or obese, and 94% had at least one chronic condition, with 44% having 3 or more. Sixty-three percent reported having hypertension, 25% COPD or asthma, 23% diabetes, and 9% heart disease.

**Table 1:** Characteristics of the sample.

**Characteristic**	**Mean**	**SD**
Age (*N* = 102)	68.98	8.58
	**Number**	**%**
**Gender (*****N*** = **102)**		
Male	30	29.41
Female	72	70.59
**Age (*****N*** = **102)**		
55-64	32	31.37
65-74	47	46.08
75+	23	22.55
**Lives alone (*****N*** = **102)**		
No	71	69.61
Yes	31	30.39
**Education (*****N*** = **94)**		
High school degree or less	33	35.11
Associate degree or some college	39	41.49
College or post-graduate degree	22	23.40
**Annual income (*****N*** = **97)**		
<$50,000	73	75.26
>$50,000	24	24.74
**Self-rated health (*****N*** = **99)**		
Fair or poor	35	35.36
Good	37	37.37
Very good or excellent	27	27.27
**Body mass index (BMI) (*****N*** = **97)**		
Healthy (<25)	23	23.71
Overweight (25– <30)	29	29.90
Obese (30+)	45	46.39
**Number of chronic conditions (*****N*** = **100)**		
0	6	6.00
1 or 2	50	50.00
3+	44	44.00

^*^Totals in the table vary due to missing data.

### 3.2. Characteristics of participant diets

Participants consumed an estimated average (±SD) of 1,533 (±650) calories per day (Table 2). Approximately 32% (±7%) of calories came from fat, with 10% (±3%) from saturated fat. Medians (inter-quartile range) for consumption of proteins, fats, and carbohydrates were 60 (46), 51 (31), and 197 (125) grams, respectively. The average total HEI score was 68, which would earn a “D” by suggested USDA grading criteria (Table 2). Only 7% of participants had a total HEI score that would earn an “A” or “B”. Based on HEI component sub scores, most participants met the guidelines for total fruit (70%) and whole fruit (78%) consumption, while 39% met the guidelines for vegetable consumption. Most participants (70%) were food secure, but 30% had some level of food insecurity. Food environment was generally favorable. The majority of participants agreed with the statements about their neighborhood: the fresh fruits and vegetables were of high quality (69%); a large selection of fresh fruits and vegetables were available (73%); and a large selection of low-fat products were available (68%).

**Table 2:** Dietary characteristics of the sample (***N*** **=** 102).

	**Median**	**IQR**
Total protein (g)	60.38	45.71
Total fat (g)	50.95	31.09
Total carbohydrates (g)	196.93	125.39
	**Average**	**SD**
Total calories (kcal)	1,532.72	650.16
% calories from fat	31.85	7.43
% calories from saturated fat	9.51	2.85
Total healthy eating index (HEI) score	67.97	8.82
	**#**	**%**
**Healthy eating index (HEI) score**		
A (90–100)	0	0
B (80–89)	7	6.86
C (70–79)	42	41.18
D (60–69)	34	33.33
F (0–59)	19	18.63
**Total fruit HEI sub score**		
Meets guideline (5)	71	69.61
Does not meet guideline (<5)	31	30.39
**Whole fruit HEI sub score**		
Meets guideline (5)	80	78.43
Does not meet guideline (<5)	22	21.57
**Vegetable HEI sub score**		
Meets guideline (5)	40	39.22
Does not meet guideline (<5)	62	60.78
**Food insecurity**		
Food secure (0)	71	69.61
Food insecure (1–6)	31	30.39
**The fresh fruits and vegetables in my neighborhood are of**		
**high quality**		
Disagree (<3)	16	15.84
Neutral (3)	15	14.85
Agree (>3)	70	69.31
**A large selection of fresh fruits and vegetables is available**		
**in my neighborhood**		
Disagree (<3)	16	15.84
Neutral (3)	11	10.89
Agree (>3)	74	73.27
**A large selection of low-fat products is available in**		
**my neighborhood**		
Disagree (<3)	17	16.83
Neutral (3)	15	14.85
Agree (>3)	69	68.32
**Average food environment score**		
Disagree (<3)	17	16.83
Neutral (3)	9	8.91
Agree (>3)	75	74.26

### 3.3. Associations between diet quality and food access (food environment and food insecurity)

#### 3.3.1. Bivariate analyses

Food insecurity was significantly associated with total HEI (*p* = 0.0052) and with the total and whole fruit HEI sub scores (*p* = 0.0090, *p* = 0.0240, respectively) (Table 3). However, food insecurity was not significantly associated with percent of calories consumed from fats, or vegetable HEI. There was no significant association between the food environment and total HEI, % of calories consumed from fats, fruit HEI, or vegetable HEI (Table 4).

**Table 3:** Bivariate associations between food insecurity and diet quality (*N* = 102).

**Diet quality**	**Food insecurity**				
	**Food secure**		**Food insecure**		
	**mean**	**SD**	**mean**	**SD**	***p*** **-value**
Total healthy eating index (HEI)	69.57	7.77	64.32	10.05	0.0052^*^
Percent calories from saturated fat	31.85	7.58	31.86	7.20	0.9953
Percent calories from fat	9.22	2.72	10.18	3.08	0.1204
	**#**	**%**	**#**	**%**	***p*** **-value**
**Total fruit HEI sub score** ^#^					0.0090^*^
Meets guideline (5)	55	77.46	16	51.61	
Does not meet guideline (<5)	16	22.54	15	48.39	
**Whole fruit HEI sub score**					0.0240^*^
Meets guideline (5)	60	84.51	20	64.52	
Does not meet guideline (<5)	11	15.49	11	35.48	
**Vegetable HEI sub score**					0.3416
Meets guideline (5)	30	42.25	10	32.26	
Does not meet guidelines (<5)	41	57.75	21	67.74	

^#^Whole fruits + juice.

^*^p <0.05.

**Table 4:** Bivariate associations between food environment and diet quality (*N* = 101).

**Agreement with availability and quality of food environment (average score)**				
**Diet quality**	**Disagree**	**Neutral**	**Agree**	***p*** **-value**
	**Mean** ±**SD**	**Mean** ±**SD**	**Mean** ±**SD**	
Total healthy eating index (HEI)	68.2 + 8.1	65.5 + 10.9	70.3 + 9.7	0.3258
Percent calories from fat	9.5 + 2.7	10.4 + 2.9	8.5 + 3.4	0.2128
Percent calories from saturated fat	31.7 + 6.8	34.0 + 7.9	30.0 + 9.9	0.3397
	**Number (%)**	**Number (%)**	**Number (%)**	***p*** **-value**
**Total fruit HEI sub score** ^#^				0.8107
Meets guideline (5)	10 (62.5)	10 (71.4)	50 (70.4)	
Does not meet guideline (<5)	6 (37.5)	4 (28.6)	21 (29.6)	
**Whole fruit HEI sub score**				0.9436
Meets guideline (5)	12 (75.0)	11 (78.6)	56 (78.9)	
Does not meet guideline (<5)	4 (25.0)	3 (21.4)	15 (21.1)	
**Vegetable HEI sub score**				0.3611
Meets guideline (5)	4 (25.0)	7 (50.0)	28 (39.4)	
Does not meet guideline (<5)	12 (75.0)	7 (50.0)	43 (60.6)	

^#^ Whole fruits + juice.

#### 3.3.2. Multi-variable analyses

After controlling for age, gender, education, living arrangement, physical health, BMI, and number of chronic conditions, we still found no significant associations between the food environment and total diet quality or diet quality components (Table 5). The significant association between food insecurity and total HEI (*p* = 0.048), and the total fruit (*p* = 0.049) and whole fruit HEI (*p* = 0.030) sub scores persisted (Table 5). Of note, none of the controlled variables were significantly associated with total HEI, vegetables, or percent calories from saturated fats. Participants who lived alone were significantly less likely to meet the guidelines for total fruits (*p* = 0.007) and whole fruits (*p* = 0.018). Compared to those with high school education or less, those with a college degree consumed significantly more calories from fat (*p* = 0.022).

**Table 5:** Multivariable^†^ associations between food insecurity and food environmentand diet quality: linear and logistic regressions (*N* = 85).

	**Diet quality**											
	**Total HEI**		**Total fruits** ^#^		**Whole fruits**		**Vegetables**		**% calories from fat**		**% calories from saturated fat**	
	**B (SE)**	***p***	**AOR (95% CI)**	***p***	**AOR (95% CI)**	***p***	**AOR (95% CI)**	***p***	**B (SE)**	***p***	**B (SE)**	***p***
**Food insecurity**												
Secure	Ref	Ref	Ref	Ref	Ref	Ref	Ref	Ref	Ref	Ref	Ref	Ref
Insecure	−4.18 (2.08)	0.048^*^	0.31 (0.10–0.99)	0.049^*^	0.22 (0.05–0.86)	0.030^*^	0.73 (0.23–2.29)	0.592	0.98 (0.67)	0.148	−0.33 (1.74)	0.849
**Food environment (agreement that food environment is good)**												
Agree	Ref	Ref	Ref	Ref	Ref	Ref	Ref	Ref	Ref	Ref	Ref	Ref
Neutral	0.97 (3.06)	0.753	0.34 (0.06–2.00)	0.234	0.37 (0.05–2.71)	0.329	0.79 (0.15–4.12)	0.783	−1.28 (0.98)	0.197	−1.50 (2.55)	0.558
Disagree	1.74 (2.75)	0.530	1.03 (0.21–5.17)	0.969	0.99 (0.15–6.41)	0.994	0.82 (0.18–3.79)	0.799	0.08 (0.88)	0.930	2.12 (2.29)	0.359

^*^P <0.05.

^#^Whole fruits + juice.

^†^Controlling for age, gender, education, living arrangement, physical health, Body Mass Index (BMI), and number of chronic conditions. None of the controlled variables were statistically associated with Total HEI, Vegetables; and % calories from saturated fats. Living arrangement (living alone) was negatively associated with Total Fruits (p = 0.007) and Whole Fruits (p = 0.018). Education was positively associated with Calories from fat (p = 0.022).

HEI, Healthy Eating Index.

B, Beta Coefficient.

SE, Standard Error.

Ref, Reference.

## 4. Discussion

Pre-pandemic studies found that older African Americans had poor overall diet quality and did not meet fruit, vegetable, and low-fat guidelines based on USDA recommendations (3, 7–9, 46–48). These findings are crucial because poor diet quality is associated with poor outcomes, including all-cause mortality (49–53). While studies on dietary habits during the COVID-19 pandemic have yielded mixed results (54–56), research specifically on older African Americans remain limited (57). Our study fills this gap by providing insights into the diet quality of older African Americans during the pandemic.

Our findings indicate that during the pandemic, older African Americans had poor diet quality. Specifically, the average total HEI score was 68, which would earn a “D” grade based on suggested USDA scoring. Only 7% of participants had a total HEI score that would earn an “A” or “B,” highlighting the pressing need for dietary improvements in this population. In contrast, over 60% of participants had fruit and vegetable scores that met the guidelines and received the maximum score of 5, which would earn an “A”. The Dietary Guidelines for Americans recommend that adults get 20–35% of their calories from fats and limit saturated fats to <10% of their caloric intake (58). While participants were close to the suggested limits for fat and saturated fat, with averages of 32.9% and 9.5% of calories coming from fat and saturated fat, respectively, they did meet the guidelines. Because the total HEI quality score is computed from all food sources, including fruits and vegetables, this finding suggests deficiencies in the other types of foods consumed.

Our findings underscore the complex and multifaceted determinants of dietary behavior during the pandemic. Our study focused on the impact of food insecurity and the food environment on diet quality. Building upon our previous work, which identified how attitudes toward COVID-19 influenced diet quality in underserved older African Americans (59), the current study expands our understanding of associations between food insecurity, the food environment, and diet quality in this vulnerable population during the pandemic.

### 4.1. Diet quality and food insecurity

Before the COVID-19 pandemic, food insecurity was already more prevalent among African Americans and had been linked to poorer diet quality (12, 19, 20). The pandemic further exacerbated this issue, with widespread reports of food insecurity among older adults, and particularly impacting racial/ethnic minority groups (28, 29, 60, 61). Dubowitz et al. (28) examined the impact of COVID-19 shutdowns on food insecurity among predominantly African American adults living in an under-resourced community. They found that despite steady declines since 2011, food insecurity increased from 21% in 2018 to 36% in 2020 and skyrocketed to 80% due to the pandemic. This heightened level of food insecurity is a critical concern, as previous research has associated food insecurity with chronic disease (3, 21, 62–65).

In previous studies, food insecurity was found to be associated with lower consumption of fruits and vegetables and recommended nutrients, as well as a higher likelihood of consuming a high-fat, and high-calorie diet (11, 14, 23, 66–69). However, our study found that food insecurity was specifically associated with lower consumption of whole and total fruits, but not vegetable or fat consumption. The observed association between food insecurity and reduced consumption of whole fruits underscores the necessity for focused interventions to enhance access to these nutritious options, which offer the added benefit of high fiber content. Moreover, our study revealed that fewer older African Americans met the guidelines for vegetable consumption compared to those meeting the guidelines for fruits, emphasizing the significance of interventions targeting vegetable intake as well. Although our study did not find a significant association between food insecurity and vegetable or low-fat food consumption, we interpret this result with caution due to our smaller sample size. Indeed, the lack of association might also be attributed to the lower efforts to meet overall dietary guidelines.

Overall, these findings underscore the critical importance of addressing food insecurity and improving access to nutritious foods, especially fruits and vegetables, to enhance the diet quality and overall health outcomes for older African Americans.

### 4.2. Diet quality and the food environment

In addition to the high prevalence of food insecurity, African Americans were already facing other challenges that contributed to poor diet quality before the pandemic, such as a lack of access to healthy food options, including fruits, vegetables and low-fat options (15, 24, 70). A study conducted by Zenk et al. (70) found that older African Americans had limited access to supermarkets and grocery stores that offer fresh and healthy food options, leading to what has been termed as “food deserts”. Interestingly, in our study, the availability or absence of healthy foods in the neighborhood did not impact the diet quality of our participants. It is worth noting that during the pandemic, local government initiatives such as the Senior Meal Emergency Response Program were put in place to increase access to food. Such efforts may have impacted participants' perceptions of food availability. However, we did not assess individuals' participation in these programs.

Our findings suggest that the mere availability of food and/or high quality foods in the neighborhood does not translate to healthy diets. This finding is consistent with previous literature (71, 72). The consumption of healthy diets is influenced by additional factors such as cost, skills, and availability of food preparation time, and transportation (11, 71, 73). A systematic review of the contribution of food prices on diet quality found that foods of lower nutritional value and lower-quality diets were generally more affordable per calorie and tended to be selected by groups of lower socioeconomic status (11). Furthermore, numerous other factors can hinder the consumption of healthy diets. In a qualitative study, African American adults noted that barriers to healthy eating included a perception of departing from the cultural heritage, lack of support from family and friends, lack of information, and preferences for the taste of foods considered unhealthy (74). Additionally, older African Americans managing hypertension reported challenges sorting through dietary advice from different sources and implementing dietary changes that would support multiple chronic conditions (75).

Addressing the complexities of the dietary behaviors of older African Americans requires a comprehensive approach that considers various factors, including affordability, accessibility, cultural influences, and social support. By understanding these multifaceted barriers, we can design targeted interventions to foster healthier dietary behaviors and improve overall health outcomes within this vulnerable population.

### 4.3. Influence of the pandemic

Restrictions during the pandemic caused disruptions in the food supply chain, reduced workforce, and shopping limitations, which affected the availability and affordability of healthy food options in some neighborhoods (76–81). Economic instability caused by the pandemic increased the risk of food insecurity for many households, which was especially pronounced in communities of color that already face higher rates of food insecurity and limited access to healthy food options due to systemic inequities and structural racism (30).

Older adults were particularly affected by the pandemic's restrictions, facing challenges in grocery shopping and limited access to fresh and nutritious foods. As a result, reliance on pre-packaged meals, often high in unhealthy ingredients, increased due to their longer shelf-life (78, 79). For older African Americans, who are more likely to live in food deserts and have limited access to healthy and affordable food options, the closure of senior centers and other community sites that provide free or reduced-price meals contributed to food insecurity. Furthermore, in the thick of the pandemic, reliance on food delivery put older African Americans at a disadvantage as they are less likely to have access to reliable internet, technology equipment and skills to make this possible (77, 82).

A systematic review by Trude et. al. (77) highlighted the barriers and facilitators of access to healthy food in low-income households during the COVID-19 pandemic. The primary barriers to equitable access were cost and limited availability of online grocery opportunities in food desserts. Although the perception of lack of control in food selection discouraged online grocery shopping, convenience and lower perceived stress were reported as benefits. However, older adults who lacked access to online grocery shopping during the extreme period of the pandemic shopped less frequently and/or relied on others to shop for them (78).

Our study shows that the diet of underserved older African Americans was far from optimal during the thick of the COVID-19 pandemic despite meeting some diet quality indices. Given the established link between poor overall diet quality and chronic disease (2, 3), this is particularly important in our cohort, which had a noteworthy prevalence of overweight or obesity (76%), hypertension (63%), COPD or asthma (25%), diabetes (23%) and heart disease (9%).

### 4.4. Limitations and strengths

Although our participants met the requirement for total fruit and total vegetable consumption, this was not enough to improve their overall diet quality. Additionally, the neighborhood availability of healthy fruits, vegetables, and low-fat products did not impact their overall diet quality. These findings should be interpreted with caution as the individual, community, and national responses to support older adults temporarily during the pandemic were not considered in this study. Additionally, our smaller sample size and convenience sample may limit generalizability. There is also a possibility of recall bias due to the self-reporting of the variables. Despite these limitations, a strength of our study is that it illuminates the comprehensive dietary quality in older African Americans, a marginalized and understudied population. Additionally, our study used validated, standardized questionnaires and a detailed dietary intake methodology. Our study emphasizes the need for further investigations into the influence of access to healthy food on diet quality post-pandemic. As one of the few studies focusing on this population, our results have implications for future interventions targeting diet quality in underserved older African Americans.

## 5. Conclusion

Factors associated with suboptimal diets in older African Americans during the COVID-19 pandemic are complex and multifaceted, encompassing both individual and environmental influences. Our research contributes valuable insights into the dietary challenges faced by this vulnerable population, highlighting the need for targeted interventions addressing access, affordability, and education on healthy food choices. As we navigate the post-pandemic era, it is imperative to recognize the importance of promoting equitable access to nutritious food options, particularly for older African Americans who are disproportionately affected by food insecurity and limited access to healthy foods.

Future research should further investigate the influence of individual and environmental factors on dietary quality among underserved older African Americans, considering broader support systems and societal responses. By understanding these multifaceted determinants, we can develop more effective strategies to improve diet quality, enhance health outcomes, and promote overall wellbeing within this vulnerable population. Addressing these disparities will require a comprehensive and collaborative approach involving policymakers, community organizations, healthcare providers, and other stakeholders.

## Data availability statement

The datasets presented in this article are not publicly available as they are still in progress. Queries and requests to access the finalized datasets should be directed to lucykibe@cdrewu.edu.

## Ethics statement

This study was approved by the Institutional Review Board of Charles R. Drew University of Medicine and Science. The studies were conducted in accordance with the local legislation and institutional requirements. The participants provided their written informed consent to participate in this study.

## Author contributions

LK: Conceptualization, Data curation, Funding acquisition, Investigation, Methodology, Project administration, Resources, Supervision, Validation, Visualization, Writing—original draft, Writing—review & editing. KS: Conceptualization, Data curation, Formal analysis, Methodology, Validation, Visualization, Writing—original draft, Writing—review & editing. MB: Methodology, Supervision, Writing—review & editing. MS: Conceptualization, Data curation, Supervision, Validation, Writing—review & editing.
